# Topics of Public Interest

**Published:** 1918-09

**Authors:** 


### TOPICS OF PUBLIC INTEREST

**British Suicides.** The male rate has declined from 157 per million population for the decade ending in 1910, to 151 in 1914, 105 in 1915, rising to 111 in 1916. The female rates were: 47, 45, 45, and 38 respectively. Increased employment and better wages are credited with the improvement.

**Incomes.** For 1916, 206 persons reported incomes of a million or more; 376 between half and one million; 6,051 between 100,000 and half a million; 115,061 between 10,000 and 100,000; 150,553, 5-10,000; 72,027, 4-5,000; 85,123, 3-4,000. According to average families, it thus appears that rather less than 2% (437,936) have incomes of $3,000 and over. Substantial increases in the numbers for each grade are reported, though this is probably due partly to greater realization of the necessity of being honest.

**Automobiles.** In spite of the war, the registration of automobiles continues to increase. 422,853 have been licensed up to July 1, in the state, against 349,040 last year. Passenger autos increased from 291,115 to 335,245. The estimated receipts from the license tax are 5 million dollars, enough to build about 200 miles of cement road. Motorcycles show a slight decrease.

**Guarantee for Buffalo General Hospital.** Owing to increased expenses, the directors have circulated guarantee blanks each covering a proportionate share of whatever deficit may occur, the liability of each share being limited to $25. Any subscriber may subscribe for as many shares as he pleases.

**Commissions for Nurses.** The Canadian Army Medical Corps will commission nurses as lieutenants. They will wear the rank insignia and give and receive salutes accordingly. It may be said that, while American nurses do not hold military rank, many officers and soldiers accord them the courtesy of a military salute.

**Donors and Receptors in Blood Groups.** An abstract previously published, omitted the diagram mentioned. A still simpler way to remember the dangers of agglutination is this: Any group may be its own donor and receptor. Donation should otherwise be from a higher to a lower number and in any possible combination of numbered groups except that there is a mutual agglutination between 2 and 3. Thus

Group 1 is the universal receptor, Group 4 the universal donor.

---

**Child Welfare Work in Buffalo.** The common council has appropriated $15,000 for equipment of nutrition clinics, care of mothers and babies, education of parents and children and prevention of still births.

---

**Trudeau Memorial.** A statue donated by former patients was unveiled at the Trudeau (formerly Adirondack Cottage) Sanatorium, Aug. 10.

---

**The American Dental Assn.** met in Chicago early in August. 6000 members were in attendance. The meeting was devoted largely to the consideration of military dentistry and dental hygiene.

---

**Rejections of Drafted Men.** 1896 of 14,870 accepted by local boards of New England, were rejected after reaching camps—12.7%.

---

**Aviation Mortality.** From Sept. 1 to July 20, 155 deaths occurred in training fields. It is calculated that there are a trifle over three deaths for every 10,000 hours of flight.

---

**The Preparedness League of American Dentists,** an organization of 15,000 dentists which was formed by Dr. J. W. Beach of Buffalo, has already given free aid to one half million men, to fit them for military service. According to the present law, only one dentist is allotted to every 1,000 men. Statistics from the front show that 20% of the men in the sick wards are there because of diseases arising from infection from diseased teeth.

---

**Col. Herbert A. Bruce** of Toronto says that the physicians have stamped out typhus and typhoid fever in the Allied Armies. That only 27 cases of typhoid fever in an army of two million men were left. He asks why women are not eligible to the Medical Reserve Corps. He said that he had been instrumental in having them admitted to the Medical Service in Great Britain.

---

**Military Casualties.** At Gettysburg, the two armies numbered together about 150,000, the Union losses being 23,190 and the Confederate 36,000, a total of nearly 60,000, 40% for three days' open fighting. Casualties of 25-30% (killed, wounded, and missing) were frequent for individual units for various 2-day conflicts of the Civil War. Of course, these percentages do not apply to the aggregate of forces including reserves, except for a few battles of unusual severity and in which practically all troops were engaged. It will be interesting to compare statistics of the present war, in which there has been no single day without casualties due to combat and in which no contest termed a battle has been of less than a week's duration, whereas very few battles of previous wars of our own country exceeded two days and none except sieges, three days, if we are not mistaken.

---

**The U. S. Public Health Service** (formerly called Marine Service and organized as a branch of the Treasury Dept. to look after the merchant marine) has for some time since the declaration of war been operated practically as a branch of the Bureau of Medicine and Surgery of the Navy. It is expected that a bill will be introduced in Congress to combine it permanently with the Naval Medical Dept. For many years, the officers have been graded and paid corresponding to the army and navy Medical officers and, more recently, have worn insignia of rank.

---

**Surgeon Probationers in the British Navy.** At the outbreak of the war, senior students of medicine were allowed to complete their course. The gradually increasing need of medical assistants has led to the enrollment of junior students in the capacity mentioned, with assignment to the navy, their duties being those of assistants and to care for minor casualties.

---

**Venereal Disease in the American Expeditionary Forces.** This has been reduced by measures already mentioned, to 47.8:1000, as against a record of about 91 in the regular army before the war and somewhat lower in troops in cantonments during the war, after the elimination of the pre-enlistment incidence. As has already been remarked, the bogy of the military venereal disease menace, about which so much was written, has proved imaginary. Rather has it been proved that the real venereal menace was one of civilian life. But, not to foster an undue degree of optimism, it should be frankly stated that the low venereal incidence of soldiers is largely due to the fact that they have too little liberty and are too tired to contract venereal disease. Army coffee may be another negative fact for, as George M. Gould pointed out several years ago, the average man uses coffee and tobacco as antidotes to each other. The soldier continues to use tobacco, he drinks extremely weak coffee whose flavor does not conduce to compensation of caffeine by increased volume of consumption. There is a quite unfounded belief among many soldiers that the coffee is doped with potassium nitrate to act as an anaphrodisiac.

---

**Gas Infection.** About 70% of gas infection is due to the Welch bacillus, at least two other bacteria being concerned in the remainder. It involves muscles especially and is predisposed to by acidosis, hence by fatigue. Intravenous or other introduction of alkalies is theoretically indicated and there is some practical corroboration of the theory.

---

**Tetanus Antitoxin** has, for some time, been used by the Germans in the form of a powder, applied on cotton to the wound. The liquid preparations have also been reduced in volume.

---

**Disinfection of Wounds by Gases,** in regard to which we have published several abstracts, is generally regarded unfavorably by military authorities.

---

**Malaria.** The weight of evidence is that the human being is the essential host and that the disease is carried over from one season to another by uncured cases. Thus efficient treatment, mainly by quinine, is not merely a matter of personal therapeutics but of state sanitation. Fifteen years ago, our Associate Editor, Prof. Pel of Amsterdam, stated that while the anopheles was common in Holland, malaria had become a rare disease owing to the thorough treatment of existing cases by quinine.

---

**Mosquito-Born Diseases.** Four have been absolutely demonstrated: Malaria (three types), Yellow fever, Dengue, Filariasis. Various other infections have been pretty well demonstrated to be at least occasionally conveyed by mosquitoes, not to mention infections carried by other insects either adventitiously or obligately.

---

**German Man Power.** Statistics of deaths and prisoners, said to have been issued by the German government are as follows: 1914, Western Front, 669,800; Eastern Front, 163,-900 (5 months). 1915, W. F., 713,461; E. F., 699,600. 1916, W. F., 901,250; E. F., 359,800. 1917, W. F., 320,450; E. F., 261,250 (5 months). Total 4,089,511. These numbers probably represent definitive losses (including permanently disabled). The more intense fighting of the present year is estimated at a million casualties, at least of which, on an average military experience, 40% would be definitive. Checked by number of divisions, comparative losses of British with an army of 2 million and allowing for the temporary disability of those who would untimately be returned to the ranks, it may be held that the total potential militia of Germany is the original total of 10% of the population plus—7 million, less 5 million, equalling 2 million. The actual present estimate is 3 million but this is substantially in agreement as the two advanced yearly classes, numbering net about half a million each, have been called into service and prisoners of the 1920 class comprise almost exactly 1/7 of all prisoners corresponding to a total strength of 3,500,000 or 2,500,000 of the normal age. From this must be deducted the losses of the last two weeks or so, so that the actual discrepancy is slight. Austria, on the same proportionate basis would have had an initial militia of 5,500,000 and a remainder of about 1,500,000. The estimate on a divisional basis was less than this at the beginning of the year and is just about a million at present. Allowing for the greater number of deaths from disease and disaffection and even defection, this estimate is reasonable in comparison with that of Germany.

---

**Gigantism.** Joseph Yawniuck died July 23, in Camden, N. J., aged 19. He was 7 feet 10 inches tall. Death was ascribed to the pituitary disease.

---

**The N. Y. Military Census** of males between 16 and 50, was 2,914,909, out of a total population of about 11 million.

---

**New Source of Alcohol.** The sotol plant which grows wild in Mexico is said to be available on a practical scale.

---

**Mobilization of the Entire Medical Profession.** We have been unable to learn the details of the proposed government measure to constitute the entire profession as a reserve medical reserve. There is no question either as to the necessity or the right of the federal government to take such action as a war measure, nor as to the general willingness of the profession to accept such regulations as may be imposed. Probably the principal duties required will be examination of recruits and an occasional compulsory change of residence to insure adequate medical attention to communities without a physician. It is possible that the plan as finally evolved may go some distance along the way of socialization of medical service but, so far as may be judged from the conduct of the railroads, it is unlikely that any radical permanent interference with private enterprise is contemplated. Now is the time for the presentation of the views of physicians themselves. Later even well meant constructive criticism must yield to obedience to orders.

---

**French Statistics of Medical Education.** From 1914 to 1917, the number of male students was, respectively 10,045, 2,944, 3,263, 3,375. Female students, 1,088, 772, 765, 822. Foreign students, 1,421, 625, 539, 515.

---

**Food Prospects.** This year's wheat crop for the U. S. will amount to nearly a billion bushels. Allowing for the proportion of the population at early and late ages, this corresponds to nearly double the total food requirements of the whole country, including the army and navy, for a year. As other cereals are also abundant, corn especially, and of nearly equal food value, and as cereals need not comprise more than half of the entire ration, we can spare enough to afford an ample cereal ration for the entire military and civil population of all of our allies whose countries are active belligerents. The abundant apple crop prophecied, though not of so great apparent importance, is also a very practical hygienic help, especially as there was a scarcity of fruits last year. The scarcity of sugar seems inevitable, though not of serious degree. Some general plan should, however, be immediately worked out, to insure the preservation of various fruits for which the addition of sugar is usually held by housewives to be essential. These fruits should be preserved anyhow, in anticipation of an available sugar supply or for use without sugar if necessary.

---

**Drugs in the Army.** Practically every state of the Union has a law which provides that those who furnish drugs to the public shall be qualified for this professional work. In our Army the hospital steward who dispenses the medicines ordered by the physician for the sick soldier is detailed from the ranks without requirement of pharmaceutical training.

To remedy this defect and thus increase the efficiency of the Medical Department of the Army, it is proposed to establish a Pharmaceutical Corps, which will be under the command of the Surgeon-General of the Army.

A bill has been introduced to provide a Pharmaceutical Corps in that department. It provides for the establishment of a Pharmaceutical Corps to be composed of a pharmacist director, with rank of major, five deputy pharmacist directors, with the rank of captain, and such number of pharmacists, with rank of lieutenant, and of pharmacist apprentices, as may be needed for the service. The bill delegates to the Pharmaceutical Corps the following duties: To procure by purchase or manufacture all supplies of medicines, drugs, chemicals, pharmaceutical apparatus, and hospital and surgical dressings necessary for the Medical Department of the Army; to determine the quality and purity of such supplies; to have charge of the medical supply depots of the Army and the storage and safeguarding of such supplies; to provide for the issuance and distribution of such supplies and the dispensing of medicines in the various hospitals, dispensaries, infirmaries, trains and camps of the Army; to properly care for, regulate the dispensing, and to systematically account for all spirituous liquors and habit-forming drugs purchased for the department; to procure by purchase or manufacture such drugs, chemicals, reagents, tests, and biologic products as are used in the laboratories and the medical and surgical practice of the department for the purpose of diagnosis, prophylaxis, or treatment; to account for all moneys received from sales of medical supplies, in accordance with the provisions of the Army regulations or disposed of by order of competent authority; to inspect the department's stores and supplies of drugs, medicines, hospital dressings, reagents, tests and biologic products and determine their deterioration and fitness for use; to co-operate with the other branches of the department in rendering first aid and wound dressing and in the making of diagnostic and chemical tests; to establish and maintain a systematic course of study and training, including the advances made in medicine, pharmacy, and sciences allied thereto, to be pursued by the members of the Army Pharmaceutical Corps who are seeking promotion in the Corps.

---

**The Public Health Service** will vaccinate against smallpox or typhoid fever, any persons, free of cost, who will call at any one of the service stations.

---

**Two Aluminum Identification Tags,** each the size of a silver half-dollar and of suitable thickness, will be worn by each officer and soldier of the American Expeditionary Forces and by all civilians attached thereto. These tags will be worn suspended from the neck, underneath the clothing, by a cord or thong passed through a small hole in the tag, the second tag to be suspended from the first one by a short piece of string or tape.

In the case of officers the tags will be stamped with the name, rank; regiment, corps, or department of the wearer and the letters "U. S." either in such form as "U. S. Infantry," "U. S. Air Service," "U. S. Tank Corps," or simply the letters "U. S. A." when an officer is not a member of an organization, corps, or department.

In the case of soldiers, the tags will be stamped with the soldier's name and the letters "U. S. A." on one side and his Army serial number on the other side. The stamping on tags previously issued will be altered as practicable to conform with this order.

This is an added means of identification.

---

**Membership in Army Medical Reserve Corps, Naval Reserve Force, or Volunteer Medical Service Corps is Urged Upon All** physicians, including women. (What provision has the government made in regard to commissions for women physicians? This is a timely question, as many women doctors would volunteer eagerly, if assured of the same standing as their men colleagues. Ed.)

---

**Every Officer,** non-commissioned officer and private will receive full pay during the period he is held as a prisoner in an alien land.

---

**The Surgeon-General** has announced that no member of the military service disabled in line of duty, even though not expected to return to duty, will be discharged from service until he shall have attained complete recovery or as complete recovery as may be expected when the nature of his disability is considered. In furtherance of this policy, physical reconstruction is defined as complete mental and surgical treatment carried to the point of maximum functional restoration, both mental and physical. To secure this result all methods recognized by modern medicine as conductive to cure will be utilized. In other words, not only the ordinary means of medicine and surgery, including all specialties, will be utilized, but also physical measures such as are employed under physiotherapy, including hydro, electro, and mechano-therapy, active exercises, indoor and outdoor games, and passive exercise in the form of massage. Provision in the form of adequate buildings and equipment for physiotherapy have been adopted in each of the hospitals.

---

**Dr. Franklin Martin** urges that physicians ineligible to the Medical Reserve Unit join the Volunteer Medical Service Corps. An insignia has been adopted for it. It is an open work shield with the caduceus and wings, surmounted by the letters V. M. S. C. This corps is not authorized to give rank. Arrangements shall be made between a member and the agency requesting service. The question of compensation and place of service, whether with or without rank, must be determined at that time. For further information, write to the Medical Section, Council of National Defense.

---

**The First Report** of the comparative cost of the operation and maintenance of the Air Mail Service shows that the airplanes used in this service have broken all records for economy of gas consumption.

The total of all operating expenses of nine airplanes covering flights aggregating 7,234 miles, was $3,682. The total consumption of gas representing 113 hours and 8 minutes of flying was 1.377 gallons, which is $32.50 per hour—something over 50 cents per mile. The total cost of gas was $405 in flying 7,234 miles.

---

**A Six Months' Supply** of bacilli Welchi serum for the cure and prevention of the deadly gaseous gangrene, or malignant gaseous edema, amounting to 120,000 doses, has been ordered by the American Red Cross in this country for shipment to the Red Cross Commission in France. The serum is a development of the last year or so and the supply is limited.

The bacilli Welchi, a small, spore-bearing germ which is the cause of gaseous gangrene, was discovered by Dr. W. H. Welch, of Johns Hopkins University, a member of the medical advisory committee of the American Red Cross, in 1892. The disease in question develops within a few hours of the time when the wound is received and, if left to itself, invariably proves fatal. Its duration is from 12 hours to 4 days. From careful anatomical studies it has been demonstrated that the disease develops only in wounds involving muscle tissue.

It is used and is in its effect the same as the antitoxin of diphtheria in the present-day treatment of the latter disease.

---

**Uniform Standards** of physical examination governing the entrance to all branches of the armies of the United States for the use of all medical officers of the Medical Department of the Army and of local and medical advisory boards under the selective service regulations have been printed and mailed.

Adherence to the new rules and regulations by the local boards will, it is expected, result in uniform examinations in all parts of the country and should prevent men physically disqualified for military service from being sent to camps. At the same time the new standards will enable local physicians to make examinations with a better understanding of the needs of the Army and will clear away misconceptions and misunderstandings which will result in the sending of men to camps who heretofore were rejected.

---

**On Representation** of responsible heads of the Red Cross, Y. M. C. A., and other allied bodies who are doing war work in France that they are unable to obtain a sufficient number of women as workers, the prohibition by the War Department, concerning the granting of passports to relatives of officers and men in the United States Army, is modified so as to permit the use of sisters of soldiers as workers under the following conditions:

First. The sisters must be duly accredited members of one of the regular authorized organizations.

Second. Each must be particularly qualified by training for the position she is to fill.

Third. That she is sent to France as a worker and not as a relative.

Fourth. That she will make no efforts to visit her relative in France, whether sick or well.

Fifth. That the organization to which she belongs will make itself responsible for returning her to America in case she violates these rules.

Sixth. That if she marries an officer or a soldier in the American Expeditionary Forces after her arrival she will automatically be sent back to the United States by the organization in which she is serving.

---

**To Safeguard the Health of American Soldiers** on the transports going to France, strict medical and sanitary precautions are taken.

Before embarking a thorough examination of troops is made by Army medical officers to eliminate the sick. Within five days of sailing the commanding officer of troops submits to the senior naval surgeon a statement that all his men have received protective vaccinations; and if any have not, he designates the men to be vaccinated.

After embarkation all troops must spend at least an hour and a half daily on deck, each man bringing his blankets to be aired. Commanding officers must see to it that their men receive 30 minutes of physical exercise during this period. The men are expected to stay in the open as much as the weather will permit.

All men and their effects must be inspected twice weekly by medical and commanding officers to detect the sick and make sure that the men are observing the rules of hygiene. The men sleep ''head and points'' to prevent, as far as possible, the spread of infection by coughing. The officers are instructed to see that the men sleep with proper coverings and that they do not sleep on deck or elsewhere unless properly protected.

Men are not permitted to close the ventilators or otherwise interfere with the flow of air. They are not permitted to eat food in berth spaces. Food is not served in rooms or other unauthorized places unless so ordered by the senior naval surgeon in case of sickness. Guards are stationed day and night at drinking fountains and other points to enforce cleanliness.

Spitting on deck is strictly forbidden. Every man must take a shower bath daily and change his underclothing at least once during the voyage.

---

**Electrical Compass.** After more than 30 years' experimenting a New Jersey physician has perfected an electrical ship's compass which records on paper all the courses taken by a vessel during a cruise of any duration.

---

**Wassermann Reaction.** H. J. Corper, of Chicago, publishes in the American Review of Tuberculosis, for July, the results of Wassermann reactions with over 2,500 sera of tuberculous patients. He reviews the more recent literature dealing with the Wassermann reaction among different groups of people, including the tuberculous. He himself reports that among 1,395 men and 1,399 women residents of the city of Chicago Municipal Tuberculosis Sanatorium, a definite positive Wassermann reaction was obtained in 7.2% of the men and 15.8% of the women.

The incidence of a definite positive Wassermann reaction as far as this could be determined, did not reveal any striking differences from the above figures resulting from a classification of the cases according to different ages or different nationality, or race.

---

**Anticigarette Reformers** would get scant consideration from the medical corps of the United States army, according to major surgeons stationed in hospitals along the front, some of whom say that the cigarette produces a relaxation for the wounded and the men just out of the trenches that no medicine could possibly produce.

"I have seen men borne in on stretchers or staggering in on their feet, with their faces contorted showing either physical pain or mental strain from their grim experiences in the trenches, relax, smile and ask for something to eat, after having a whiff of a cigarette," said a surgeon in charge of a casualty clearing station.

"The effect of the cigarette is wonderful. It certainly is not medicinal, for the action is too quick. As soon as the lads take their first whiff they seem eased and relieved of their agony."

**Bruck Test.** H. J. Corper and L. Fiala, of Chicago, report in the July number of the American Review of Tuberculosis, on the serochemical reaction of Bruck, which they tried on 228 sera in a tuberculosis sanatorium. They found this reaction for syphilis unreliable as a test to supplement the Wassermann reaction in the tuberculosis sanatorium. Of 14 Wassermann reacting sera six gave a negative serochemical reaction. Of 213 negative Wassermann reaction sera, 113 gave a positive Bruck test. The percentage of positive was greater among the moderately advanced and far advanced cases of tuberculosis than among other classes of tuberculosis cases and of non-tuberculous cases.

**Centenarian.** Rev. Albert Vogel of Jeannette, Pa , a native of Bavaria, who came to this country in 1829, and who served in the Civil War, is still active and said to have the appearance of a man of 60 at the age of 101. He has been and still is preaching, for a period of 85 years. He had 11 children of whom 6 survive; 17 grandchildren, 34 great-grandchildren, 3 great great-grandchildren. John Dempsey of Marion, Ill., died in June 3 days before his 100th birthday. He had been married 13 times, the last time 5 years ago. Wm. Masios, a negro preacher, died at the Bellevue Hospital, N. Y. City, Aug. 15, claiming to have been born in Richmond, Vt., in 1771. His answers to questions as to the times in which he lived were so intelligent as to support his claim. The Medical Review of Reviews, July, mentions a Mrs. Gold of Brooklyn as living at 102, able to do her own washing and presumably other forms of housework. She has 2 children, 14 grandchildren and 8 great-grandchildren.

